# Supplementary material for: Stabilization of Natural Pigments in Ethanolic Solutions for Food Applications: The Case Study of Chlorella vulgaris
Source: Molecules. 2023 Jan 3;28(1):408. doi: 10.3390/molecules28010408 (PMC9822436; doi:10.3390/molecules28010408)
Supplement: Supplementary file 1 [file molecules-28-00408-s001.zip › molecules-2057901-supplementary.pdf]

molecules

MDPI

Supplementary materials

Stabilization of natural pigments in ethanolic solutions for food applications: the case study of *Chlorella vulgaris*

Andreia S. Ferreira [1], Liliana Pereira [1], Feliciana Canfora [1], Tiago H. Silva [2,3], Manuel A. Coimbra [1], and Cláudia Nunes [4,*]

1 LAQV-REQUIMTE, Department of Chemistry, University of Aveiro, 3810-193, Aveiro, Portugal

2 3B´s Research Group, I3Bs – Research Institute on Biomaterials, Biodegradables and Biomimetics of University of Minho, Headquarters of the European Institute of Excellence on Tissue Engineering and Regenerative Medicine, AvePark – Parque de Ciência e Tecnologia, Zona Industrial da Gandra, 4805-017 Barco, Guimarães, Portugal

3 ICVS/3B's - PT Government Associate Laboratory, Braga/Guimarães, Portugal

4 CICECO–Aveiro Institute of Materials, Department of Materials and Ceramic Engineering, University of Aveiro, 3810-193 Aveiro, Portugal

* Correspondence: claudianunes@ua.pt

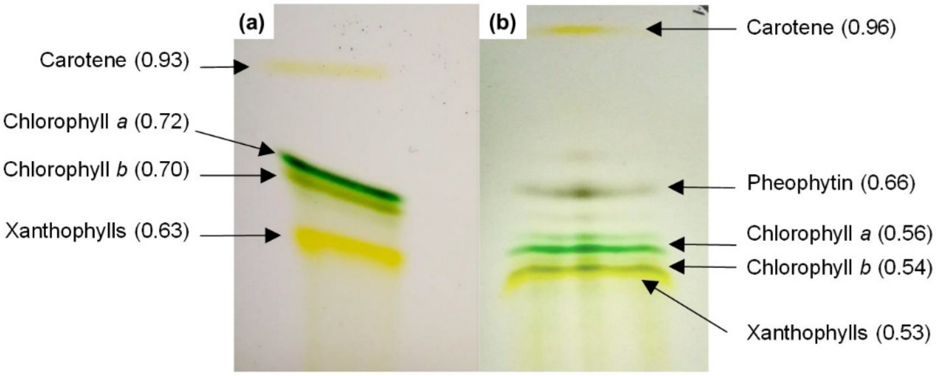

Figure S1 - TLC of ethanol *C. vulgaris* extract with two different eluents (a) petroleum ether:1-propanol:water (100:10:0.25, v/v/v); (b) *n*-hexane:acetone (7:3, v/v). The number between parentheses corresponds to the Rf value.

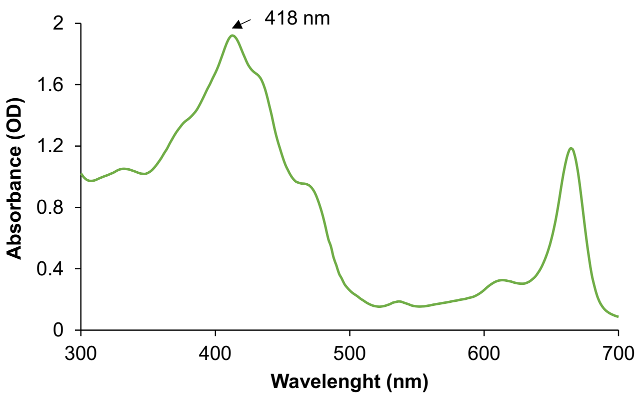

Figure S2 – Ultraviolet-visible spectrum of *C. vulgaris* ethanolic extract.

Table S1 - Absorbance at 418 nm (Y_1) and -a* (Y_2) settled according to full factorial design for 48 h of *C. vulgaris* ethanolic extract.

Run	X_1 (T, ºC)	X_2 (Light)	X_3 (Atm)	X_4 (Alkaline)	Y_1 (A418)	Y_2 (-a*)
1	-1 (4)	-1 (Dark)	-1 (Air)	-1 (No NaOH addition)	0.444	8.65
2	+1 (60)	-1 (Dark)	-1 (Air)	-1 (No NaOH addition)	0.258	2.78
3	-1 (4)	+1 (Light)	-1 (Air)	-1 (No NaOH addition)	0.247	2.58
4	+1 (60)	+1 (Light)	-1 (Air)	-1 (No NaOH addition)	0.129	0.96
5	-1 (4)	-1 (Dark)	+1 (Argon)	-1 (No NaOH addition)	0.429	9.26
6	+1 (60)	-1 (Dark)	+1 (Argon)	-1 (No NaOH addition)	0.296	3.92
7	-1 (4)	+1 (Light)	+1 (Argon)	-1 (No NaOH addition)	0.287	4.67
8	+1 (60)	+1 (Light)	+1 (Argon)	-1 (No NaOH addition)	0.075	0.91
9	-1 (4)	-1 (Dark)	-1 (Air)	+1 (NaOH addition)	0.402	12.00
10	+1 (60)	-1 (Dark)	-1 (Air)	+1 (NaOH addition)	0.289	3.66
11	-1 (4)	+1 (Light)	-1 (Air)	+1 (NaOH addition)	0.226	3.99
12	+1 (60)	+1 (Light)	-1 (Air)	+1 (NaOH addition)	0.088	2.03
13	-1 (4)	-1 (Dark)	+1 (Argon)	+1 (NaOH addition)	0.512	10.05
14	+1 (60)	-1 (Dark)	+1 (Argon)	+1 (NaOH addition)	0.300	4.52
15	-1 (4)	+1 (Light)	+1 (Argon)	+1 (NaOH addition)	0.262	2.85
16	+1 (60)	+1 (Light)	+1 (Argon)	+1 (NaOH addition)	0.098	2.63

Table S2 - Absorbance at 418 nm (Y_1) and -a* (Y_2) settled according to full factorial design for 9.5 and 65.5 h of *C. vulgaris* ethanolic extract (data set 2).

Run	X_1 (T,ºC)	X_2 (Light)	X_3 (Alkaline)	9.5 h		65.5 h	
				Y_1 (A418)	Y_2 (-a*)	Y_1 (A418)	Y_2 (-a*)
1	-1 (4)	-1 (Dark)	-1 (No NaOH addition)	1.846	21.19	1.624	17.45
2	+1 (28)	-1 (Dark)	-1 (No NaOH addition)	1.852	21.04	1.556	17.07
3	-1 (4)	+1 (Light)	-1 (No NaOH addition)	1.463	21.40	0.893	3.02
4	+1 (28)	+1 (Light)	-1 (No NaOH addition)	1.191	15.71	0.737	0.09
5	-1 (4)	-1 (Dark)	+1 (NaOH addition)	1.833	20.72	1.679	16.69
6	+1 (28)	-1 (Dark)	+1 (NaOH addition)	1.822	20.58	1.631	16.64
7	-1 (4)	+1 (Light)	+1 (NaOH addition)	1.434	11.37	0.876	5.75
8	+1 (28)	+1 (Light)	+1 (NaOH addition)	1.229	9.48	0.727	3.72

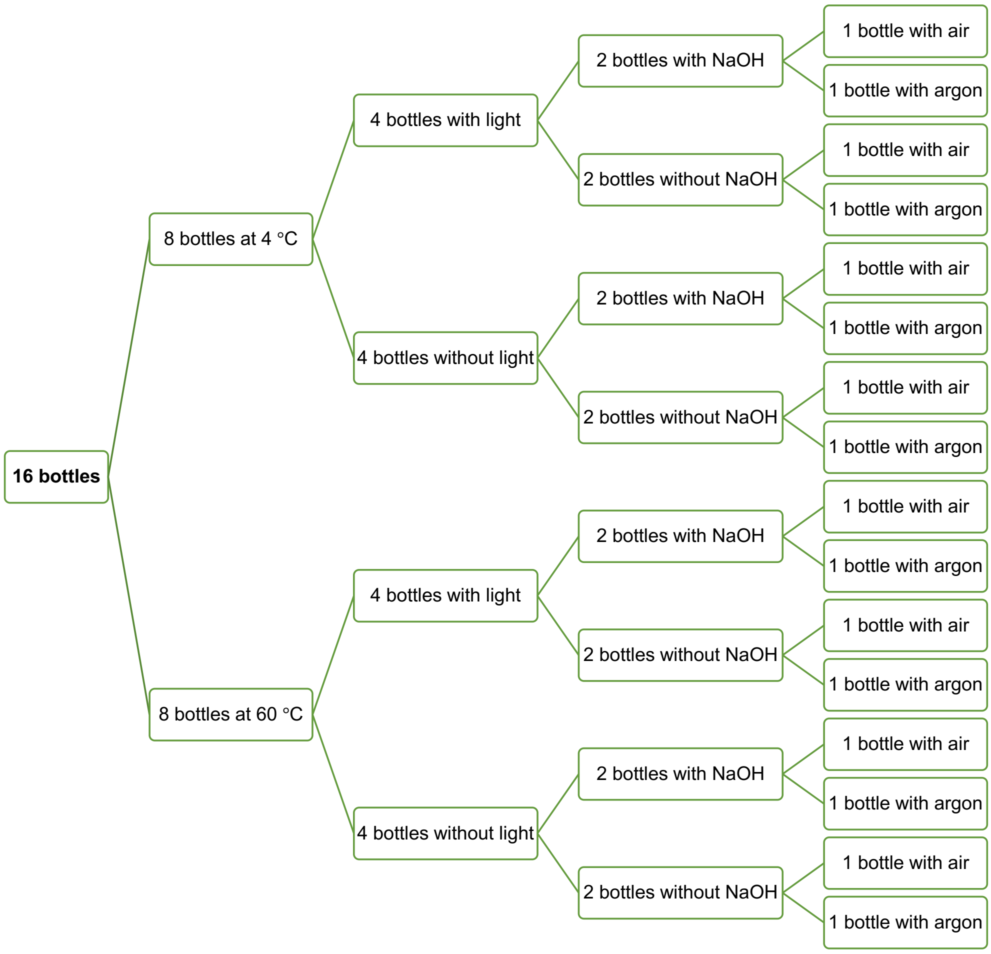

Figure S3 - Schematic representation of the study of the stability of *C. vulgaris* pigments at different storage conditions for data set 1.

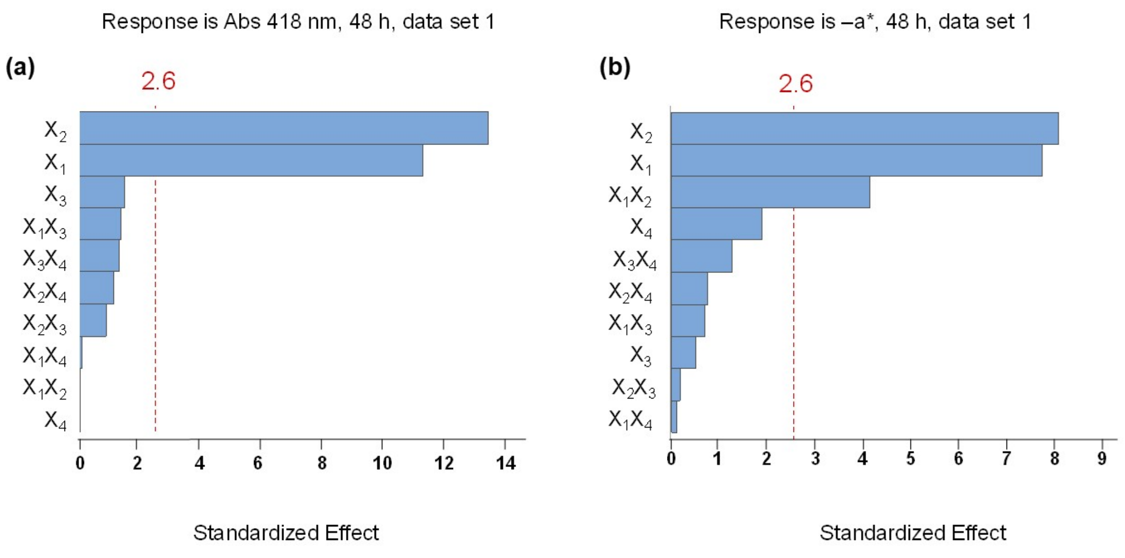

X_1- Temperature **X_2**- Light; **X_3**- Modified atmosphere; **X_4**- Alkaline

Figure S4 - Pareto charts of the standardized effects for the data set 1: (a) response is Abs (418 nm), 48 h (p<0.05); (b) response is -a*, 48 h (p<0.05).

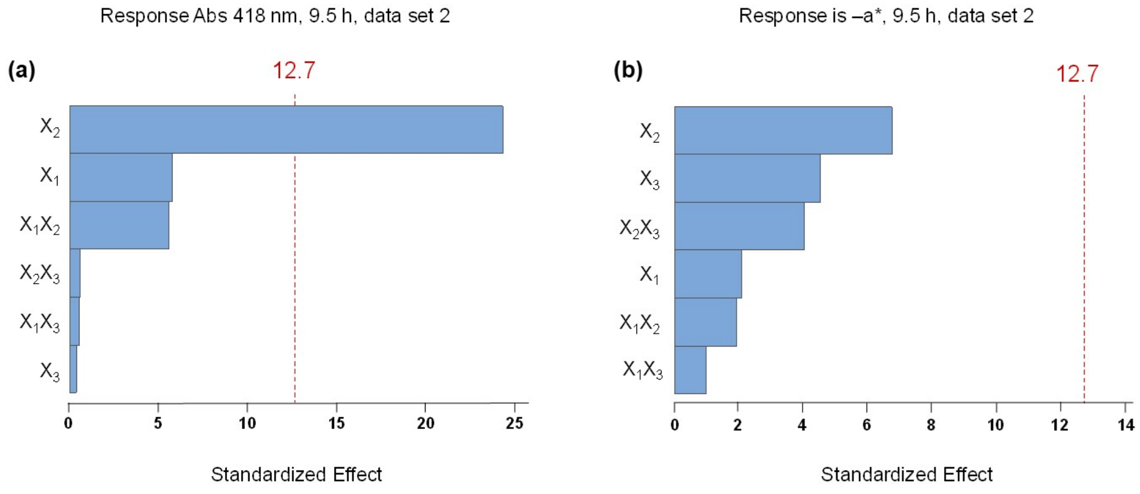

X_1- Temperature **X_2**- Light; **X_3**- Alkaline

Figure S5 - Pareto charts of the standardized effects for the data set 2: (a) response is Abs (418 nm), 9.5 h (p<0.05); (b) response is -a*, 9.5 h (p<0.05).

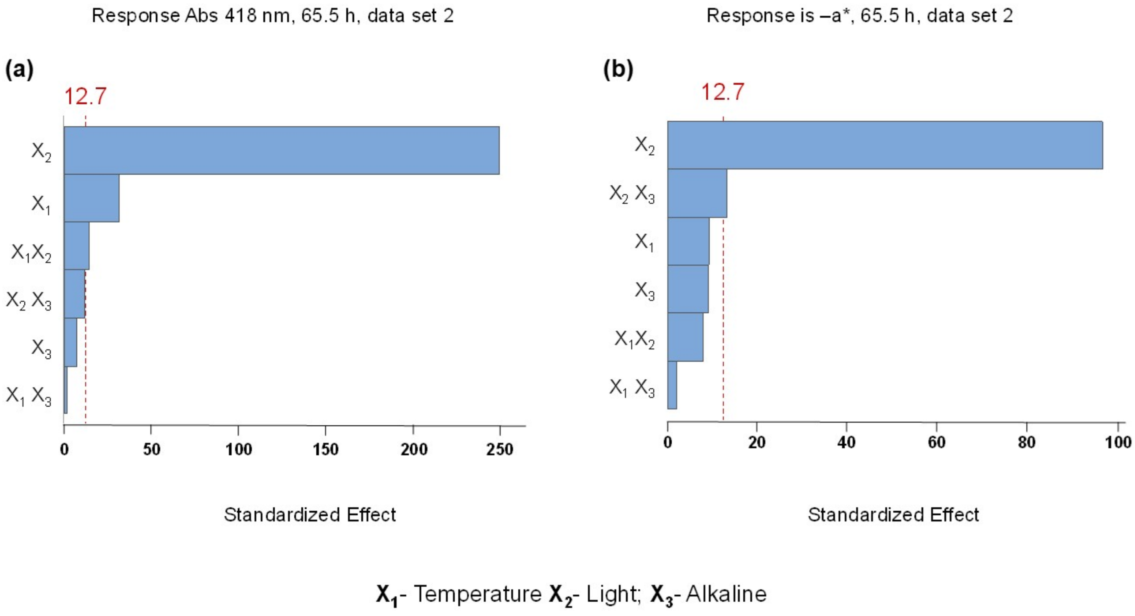

Figure S6 - Pareto charts of the standardized effects for the data set 2: (a) response is Abs (418 nm), 65.5 h (p<0.05); (b) response is -a*, 65.5 h (p<0.05).

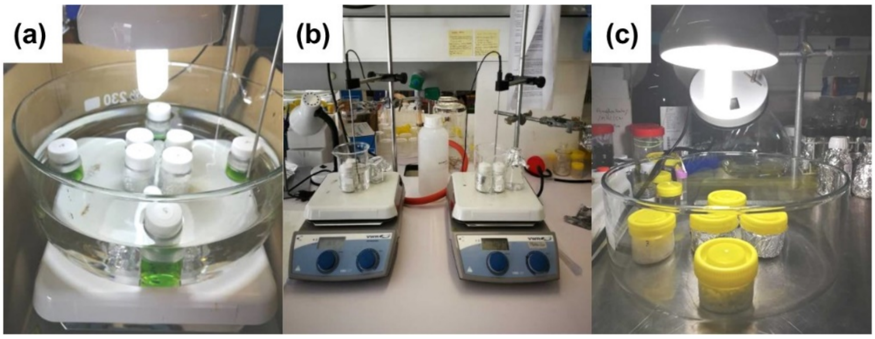

Figure S7 – (a) Evaluation of *C. vulgaris* pigments storage stability carried out at 28 °C; (b) Evaluation of kinetic degradation of green color at 45 °C and 60 °C in a paraffin bath; (c) Evaluation of color degradation of green cooked rice (with the incorporation of *C. vulgaris* ethanolic extract).
